# Review of Seismic Risk Mitigation Policies in Earthquake-Prone Countries: Lessons for Earthquake Resilience in the United States

**DOI:** 10.1080/13632469.2021.1911889

**Published:** 2022

**Authors:** Yating Zhang, Juan F. Fung, Katherine J. Johnson, Siamak Sattar

**Affiliations:** a Applied Economics Office, National Institute of Standards and Technology, Gaithersburg, Maryland, USA; b Earthquake Engineering Group, National Institute of Standards and Technology, Gaithersburg, Maryland, USA

**Keywords:** Seismic retrofit policy, earthquake resilience, building safety, hazard mitigation, best practice, incentive strategy

## Abstract

This article reviews the current state of practice in seismic risk mitigation, focusing on policies in ten of the most earthquake-prone countries around the world. In particular, the review compares policies to retrofit existing buildings and mechanisms for financing seismic risk mitigation, within the context of seismic risk and design standards for each country. The goal of the review is to identify policy best practices that may be useful for national and local governments that are interested in improving their earthquake resilience. The result is a set of best practice recommendations that are organized conceptually around key stages of the seismic retrofit process: (1) risk assessment; (2) knowledge transfer; (3) setting targets; (4) implementation; and (5) monitoring. While these lessons may be valuable to any earthquake-prone country, the recommendations are framed with particular attention to the United States where seismic risk mitigation is primarily the responsibility of local governments.

## 1. Introduction

Buildings across the world are susceptible to damage or collapse from an earthquake event, contributing to casualties and economic losses (Egbelakin, Wilkinson, and Ingham 2014). Studies have shown that the collapse of buildings is the main reason for excessive deaths and injuries during earthquakes (Coburn, Spence, and Pomonis 1992; Kenny 2009). In the early 20^th^ century, countries such as Italy, Japan, and the United States began developing earthquake-resistant design standards for new construction in response to catastrophic earthquakes (Holmes et al. 2008). Yet many of the world’s buildings were constructed prior to modern seismic design codes and standards as both knowledge of seismic risk and design standards continue to evolve (Jaiswal and Wald 2008). Moreover, buildings damaged in previous earthquakes may not be able to sustain additional damage caused by future earthquake events (Georgescu et al. 2018). Thus, there is growing appreciation for pre-earthquake risk mitigation to address the existing building stock (Filippova and Noy 2020).

This study compares seismic retrofit policies across some of the most earthquake-prone countries in the world. The goal is to identify policy best practices that may be useful for national and local governments interested in pursuing or enhancing their approach to seismic risk mitigation. While all earthquake-prone countries may find these best practices useful, the recommendations are framed with particular attention to the United States where seismic risk mitigation is primarily the responsibility of local governments. Retrofit policies are compared with respect to the criteria and requirements for retrofit, strategies for promoting retrofits, policy effectiveness, and implementation challenges. Background information on seismic risk and the evolution of design standards is presented in order to properly contextualize each country’s approach to seismic risk mitigation.

Japan, New Zealand, and the west coast of the United States are located on the circum-Pacific seismic belt, along which 90% of the world’s earthquakes (and 81% of the world’s largest earthquakes) have occurred (OCHA 2014). China, India, and Nepal frequently experience earthquakes in the Himalayan region, where mountains formed 50 million years ago and continue to grow today. Iran is located on the Alpide earthquake belt, also called the Alpine-Himalayan orogenic belt, which is the second most seismically active region in the world, after the circum-Pacific seismic belt (Hayes et al. 2020). The seismicity of European countries is not as high as that of the Pacific coast of the Americas or some Asian countries. However, earthquakes have caused substantial damage in Europe because of the large number of older buildings there. The highest earthquake hazard is concentrated in the south-eastern areas of Europe, such as Italy, Greece, Romania, and Turkey (Giardini et al. 2013).

Older buildings are typically constructed using adobe or masonry without reinforcement, using concrete with insufficient reinforcing steel, using steel with deficient connections, or using wood frames with open ground floors, which make them prone to failure when there is ground shaking. This situation has been improved as new seismic codes were developed to provide higher standards. However, the shortage of technical manpower, the deficiency in regulatory mechanisms to ensure that new construction is fully compliant with seismic codes, and economic constraints may still exist in some countries and lead to inadequate earthquake-resistant capacities of newly constructed buildings.

Seismic retrofitting does not usually make older buildings as strong as new ones. Standards typically require that retrofitted buildings have enough capacity to remain standing so occupants can safely evacuate, but those buildings may be damaged beyond repair (ICC 2017a). Current codes for most new buildings target preserving the lives of occupants by minimizing the likelihood of building collapse under significant shaking, i.e., the maximum considered earthquake (ICC 2017b). Some buildings, such as hospitals, may be subject to a higher standard so they can remain operational after a moderate earthquake, i.e., the design-level event.

### 1.1. Overview of Retrofit Policy in Selected Countries

Table 1 compares seismic retrofit policies currently implemented by the selected countries. Across several countries, the highest priority for retrofit is given to critical buildings (e.g., hospitals, emergency control centers), lifeline infrastructure, and buildings that house a large number of people (e.g., schools) and can cause massive fatalities if they collapse. At present, only a few countries (e.g., the United States, New Zealand) have jurisdictions requiring mandatory retrofit for privately-owned residential buildings, because a mandatory policy is relatively difficult to enforce and costly to implement. Most countries encourage voluntary retrofit options, and these governments often provide financial and technical assistance to building owners.

Although strengthening older buildings helps protect life and property, most building owners are reluctant to make such changes. A seismic retrofit is a major financial investment with no immediate returns on which to capitalize and the value of such an depends on the building’s ability to generate future returns (Markhvida and Baker 2018). Moreover, due to challenges in communicating hazard risk, building owners may believe that a strong earthquake will not occur during their own lifetime (Egbelakin, Wilkinson, and Ingham 2014) or that their building is not susceptible to earthquake risk (Holmes et al. 2008). Appropriate incentive policies can motivate building owners to perform seismic upgrades and thus reduce seismic risk in their communities. Table 1 further demonstrates the incentive strategies employed by the study countries. The major incentive methods are retrofit grants, tax reimbursements, and government loans. International loans and donations are utilized by a few countries.

## 2. Comparison of Retrofit Policies

### 2.1. United States

The States of California, Oregon, and Washington have the highest risk of earthquake damage in the United States, accounting for 80% of annual nationwide earthquake losses (Jaiswal et al. 2015). Out of all the U.S. states, California has had the most recent experience with damaging earthquakes as will be discussed in more detail below. Over a hundred faults run through Southern California alone. With more than 20 million inhabitants, a population size comparable to Romania, Southern California is highly vulnerable to earthquakes compared to other regions in the country (SEAOSC 2016).

In contrast to other earthquake-prone countries, seismic risk mitigation policy in the United States is primarily the responsibility of state and local governments. This is because there is no federal mandate for building codes and standards to be adopted across all jurisdictions. Building codes, such as the International Building Code (IBC) and the International Existing Building Code (IEBC) are the foundation for many building codes and standards across the U.S. (ICC 2017a, 2017b). However, these must be applied and enforced at the state or local level, meaning that there is significant variation across U.S. jurisdictions in the level of protection that may be provided by buildings in an earthquake. Nevertheless, federal agencies, such as the Federal Emergency Management Agency (FEMA), and programs, such as the National Earthquake Hazards Reduction Program (NEHRP), provide crucial leadership and guidance for local policy makers. In turn, regional initiatives can influence federal guidelines and provisions, as discussed below.

#### 2.1.1. Seismic Risk Mitigation Policies

Retrofit policies in U.S. jurisdictions largely target buildings based on structural properties. Thus, the discussion that follows is broken down by building construction type. A notable exception is California Senate Bill (SB) 1953, the 1994 amendment to the Hospital Facilities Seismic Safety Act of 1983 that mandated the seismic retrofit of hospitals in California (OSHPD 2020). SB 1953 is relatively unique in targeting buildings by occupancy, and was a direct response to the 1994 Northridge Earthquake (Preston et al. 2019). It is also notable for setting both structural and nonstructural performance targets, both of which are necessary to ensure post-earthquake hospital functionality (Jacques et al. 2014; OSHPD 2020).

##### 2.1.1.1. Unreinforced Masonry Buildings.

Unreinforced masonry (URM) bearing wall buildings have long been recognized as the most prone to damage structure type in earthquakes. After the 1933 Long Beach Earthquake (M 6.4), the California State Legislature passed the Field Act, which banned the construction of new URM buildings (FEMA 2009). However, the existing URM buildings were prone to collapse and caused considerable property loss and deaths in the 1971 San Fernando Earthquake and 1983 Coalinga Earthquake. In 1986, the California state government enacted a law requiring all jurisdictions in Seismic Zone 4 to identify potentially hazardous URM buildings and develop a risk mitigation program (Seismic Safety Commission 1992). Seismic Zone 4 represents the highest seismicity zone according to the 1997 Uniform building Code, and includes the major metropolitan areas of Los Angeles and San Francisco.

The City of Long Beach passed a mandatory ordinance to strengthen URM buildings shortly after the 1971 San Fernando Earthquake (Seismic Safety Commission 1992). The ordinance was amended in 1977 and 1990 because the earlier retrofit standards were based on requirements for new construction, resulting in substantially higher demolition rates and retrofit costs. Moreover, the earlier ordinance addressed the most dangerous and intermediate dangerous URM buildings, while the least hazardous buildings were left out. The City of Los Angeles enacted the Earthquake Hazard Reduction Ordinance in 1981, titled Division 88, applying to all URM buildings constructed before 1934. The ordinance divided buildings into several tiers and required retrofit work to be completed in 10–15 years (Seismic Safety Commission 1992). Following the state URM law passed in 1986, 52.4% of jurisdictions in Seismic Zone 4 established mandatory strengthening programs, 14.6% of jurisdictions initiated voluntary strengthening programs, and 18.0% of jurisdictions only notified building owners of potential risks (Seismic Safety Commission 1995).

In the U.S. state of Washington, the risk of a catastrophic earthquake was not known until the 1990s, when research that uncovered the massive Cascadia Earthquake of 1700 revealed the risk (Schulz 2015). As the public gradually became aware of the risk from the Cascadia subduction zone, the state and local governments began to take action. The City of Seattle established an URM Policy Committee in 2012 to develop recommendations for Seattle’s Department of Construction and Inspections on a mandatory URM seismic retrofit program. The recommendations were published in 2017 and recognized retrofit cost as a major impedance to seismic upgrade (SDCI 2017). In 2018, the city engaged the National Development Council, a private nonprofit organization, to identify potential financing and funding mechanisms and to develop strategies for mitigating financial impacts of seismic retrofit on property owners (NDC 2019). At present, the city requires seismic retrofit for URM buildings that are undergoing a major improvement or alteration. A retrofit policy applied to all URM buildings is still under development (NDC 2019).

##### 2.1.1.2. Soft-Story Buildings.

Soft-story buildings are weak at the ground level due to large openings in perimeter walls for garage doors and store windows and few interior partition walls. After the 1989 Loma Prieta Earthquake (M 6.9) and the 1994 Northridge Earthquake (M 6.7), which left thousands of housing units uninhabitable, soft-story buildings were recognized as hazardous. In 2005, the California State Legislature amended the Health and Safety Code and expressly authorized cities to adopt seismic retrofit standards that comply with nationally recognized codes through ordinances. The Code also identified wood-frame, multi-unit residential buildings, constructed before January 1, 1978, with soft, weak or open-front wall lines as potentially hazardous buildings. In 2003, the first edition of the IEBC was published, providing guidelines for “Earthquake Hazard Reduction in Existing Wood-Frame Residential Buildings with soft, weak, or open-front walls.” The Code was amended in a three-year cycle and used as the model code for seismic retrofit in California (ATC 2010).

The City of Fremont, California, adopted an ordinance in 1999 to establish retrofit standards and to have owners notified of potential earthquake hazards associated with their soft-story apartment buildings. The ordinance allows for twelve months of voluntary compliance. The retrofit standards of Fremont formed the basis of the guidelines provided by the 2003 IEBC. In 2007, the city made the ordinance mandatory for apartment buildings containing soft or open wall lines, intending to strengthen remaining buildings (City of Fremont 2007). The City of San Francisco conducted a range of studies on earthquake resilience after the 1994 Northridge Earthquake, and found that existing engineering procedures were not adequate to fully evaluate the complex behavior of vulnerable buildings and were not necessarily yielding optimal retrofit designs (ATC 2010). In 2012, the Federal Emergency Management Agency (FEMA) released the *P*-807 method to provide detailed procedures on seismic analysis and retrofit for vulnerable wood-frame buildings (ATC 2013). The guidelines limited the structural retrofit work to the first story and second-floor diaphragm in order to improve building performance while limiting retrofit costs. The City of San Francisco established a mandatory strengthening program for multi-unit wood-frame residential buildings in 2013 based on FEMA *P*-807 (City of San Francisco 2013). Subsequently, the Cities of Berkeley, Los Angeles, Santa Monica, West Hollywood, Beverly Hills, Oakland, and Pasadena each adopted an ordinance to mandate seismic retrofit for soft-story buildings.

##### 2.1.1.3. Non-Ductile Concrete Buildings.

In California, many non-ductile concrete buildings were constructed before the 1976 Uniform Building Code came out, which prevented insufficient steel inside concrete columns and beams. However, the 1994 Northridge Earthquake caused severe damage to apartment buildings and hospitals that were designed even after the 1976 code. Inadequate strength in the anchorage system of the wall to the roof and floor diaphragm was found to be the major reason. Thus, new provisions were introduced into the 1997 Uniform Building Code to increase wall anchor design forces in concrete tilt-up buildings, accompanied by stricter detailing and inspection requirements.

Three weeks after the Northridge Earthquake, the City of Los Angeles passed an ordinance that provided systematic procedures and standards for identification and classification of tilt-up concrete wall buildings and mandated seismic strengthening for those buildings in three years. In 2015, the city adopted a comprehensive ordinance to mitigate earthquake hazard for non-ductile concrete buildings. The time limit for compliance varies by the risk level of the building and is up to 25 years (City of Los Angeles 2015). The City of Santa Monica adopted a series of emergency ordinances after the Northridge Earthquake to align procedures for the restoration or demolition of damaged buildings, and to establish evaluation and retrofitting standards for public buildings, critical facilities, and hazardous constructions. The city amended the ordinance in 1999 to address all existing concrete buildings, and revised it again in 2017 to mandate seismic diagnosis and retrofit (City of Santa Monica 2017). Other cities that have developed a strengthening program for concrete buildings include Burbank, Fremont, and West Hollywood.

##### 2.1.1.4. Steel Moment-Frame Buildings.

Steel moment-frame buildings were believed to be one of the most seismic-resistant structure types. However, in the 1994 Northridge Earthquake, unexpected damages occurred in welded beam-to-column moment connections, although the connection failures did not lead to collapse or casualties (SAC 1995). The Interim Guidelines FEMA-267, published in 1995, detailed procedures and standards for evaluation, repair, modification, and design of steel moment frames. The guidelines not only supported repair work for damaged structures but also improved seismic design for new construction (SAC 1995). In 2000, FEMA published new guidelines for steel moment-frame buildings as FEMA-350 through FEMA-353, with FEMA-351 focused on seismic evaluation and upgrade criteria for existing buildings (SAC 2000).

The 1994 Northridge Earthquake also motivated the City of Los Angeles to pass an ordinance that required building owners to submit a report indicating the number of damaged welded connections and proposed repair procedures within 180 days. For buildings that suffered damage, owners were responsible for obtaining permits within 90 days and completing repairs within two years (City of Los Angeles 1995). In 2017, the City of Santa Monica passed an ordinance that required owners to perform seismic diagnosis and retrofit for all existing steel moment-frame buildings in 20 years (City of Santa Monica 2017). The City of West Hollywood followed this practice and established a mandatory retrofit program for existing steel moment-frame buildings later the year (City of West Hollywood 2017).

#### 2.1.2. Mechanisms for Financing Seismic Risk Mitigation

In the United States, retrofits are the responsibility of the building owner. However, there are some programs that provide financial support. California began a pilot program in 2013, called the Earthquake Brace and Bolt (EBB) Program, in which the State provides a grant of $3,000 to homeowners in a few designated areas that are at high risk for earthquakes. The program has been expanded to include more than a hundred zip code areas to date. Eligible houses are made of wood frames with cripple walls (CEA 2019a). Since 2017, a new type of financing approach, called Property Assessed Clean Energy (PACE), is available in California to assist property owners to complete seismic upgrades. The program provides 100% financing, covering permits, inspections, design, and construction fees, for all kinds of buildings. The repayment period ranges from 5 to 30 years, and a low interest rate of 6.5 to 8.5% is applied (Hui 2017). Moreover, the California Earthquake Authority offers up to a 25% discount on earthquake insurance premiums for wood-frame single-family homes that have been retrofitted to meet the California Existing Building Code (CEA 2019b).

In addition to state-level incentives, cities in California have employed various financial measures to promote seismic retrofit. The City of Almeda offers up to 100% fee waiver for engineering report review and construction permits for soft-story buildings. The City of Berkeley provides a retrofit grant that covers up to 75% of design and 40% of construction costs for non-ductile or tilt-up concrete buildings and soft-story buildings with three or four units. URM and soft-story buildings with five or more units can receive a grant that covers up to 75% and 30% of design and construction costs, respectively. The Cities of Fremont and Pasadena waive plan review and building permit fees and construction taxes as they have relatively few soft-story buildings. The City of San Francisco allows soft-story building owners to pass 100% of total seismic retrofit cost to tenants over 20 years, whereas the City of Los Angeles limits the amount to 50% of retrofit cost, passed to tenants over 10 years.

### 2.2. New Zealand

New Zealand is located along the boundary between the Indo-Australian Plate and the Pacific Plate. Every year over 15,000 earthquakes are detected in New Zealand, and about 100 to 150 of these quakes are large enough to be felt. Historical trends and records suggest that New Zealand would experience several magnitude 6 earthquakes every year, a magnitude 7 earthquake every decade, and a magnitude 8 earthquake every century (IGNS 2020).

Similar to the U.S., seismic design standards and retrofit policies are adopted at the local jurisdiction level. However, unlike the U.S., the national government can mandate adoption or development of a code or standard. After the 1931 Napier Earthquake (M 7.7), New Zealand developed its first seismic code. By 1979, a patchwork of building codes and bylaws was administered across several layers of central and local government (Buckett 2014). The Building Act 1991 established New Zealand’s first cohesive national building code: the 1992 Building Code, a performance-based code loosely modeled on Norway’s code (Buckett 2014). The Building Act 2004, which required that territorial authorities develop earthquake risk mitigation policies, requires that all building work comply with the Building Code (MacRae, Clifton, and Megget 2011).

#### 2.2.1. Seismic Risk Mitigation Policies

The Building Act 2004, the first regulation addressing existing buildings, recommended that the minimum level for seismic retrofit of existing buildings should be 34% of the code requirement for a new building standard (denoted 34% NBS). The Building Code for new construction is contained in the Act. A 34% NBS represents the minimum requirement necessary for ensuring safety in an earthquake event. A score lower than 34% NBS indicates high risk, while a score greater than 34% NBS implies moderate risk. The New Zealand Society for Earthquake Engineering argues that 67% NBS is a more suitable minimum level for seismic retrofit, because the Act’s minimum requirement (34% NBS) is inadequate to eliminate non-ductile failure in earthquake-prone buildings. A score of 67% NBS or above is typically considered a minor risk (Egbelakin 2013). Depending on the %NBS target, proper restraints for nonstructural elements may be required, as stipulated in the Building Code and supporting standards (NZS 4219:2009 and NZS 1170.5:2004).

The Building Act 2004 also stipulated that altering or changing the use of an existing building should comply with the Building Code for new construction. Moreover, local or territorial authorities were mandated to develop a policy to mitigate earthquake risks for their jurisdictions. By 2013, 73% of territorial authorities adopted seismic retrofit levels between 33% and 67% NBS, and 27% of them approved 67% NBS. In addition, 45% of local authorities chose an active approach to implement retrofit policies, 32% of them used a passive approach, and 23% of them employed a combined active-passive approach. The active approach includes a rigorous identification and detailed assessment of potential hazardous buildings and time limits for retrofitting or demolishing those buildings. The passive approach requires structural strengthening when alternating or changing the use of an existing building (Egbelakin 2013).

The Christchurch Earthquake (M 6.2) in February 2011, part of the 2010–2011 Canterbury Earthquake Sequence (CES), resulted in 185 fatalities and substantial damage due to its proximity to the central business district (He et al. 2019). The CES, which resulted in damage to 100,000 residential properties and displacement of over 300,000 people, led to the development of the Building (Earthquake-prone Buildings) Amendment Act 2016. The Act tightened requirements for the seismic retrofit of existing buildings and shortened the time frame for mandatory compliance. Earthquake-prone buildings were defined as those with an insufficient design capacity to withstand a moderate earthquake. Specifically, unreinforced masonry buildings, pre-1976 buildings with three or more stories, and pre-1935 buildings with one or two stories are considered to be potentially earthquake-prone (MBIE 2017). Three risk categories (low, medium, and high) were adopted by the Act to set corresponding deadlines for assessment and seismic upgrade. The Act applied to the whole country, but implementation (e.g., mandatory or voluntary) is determined by local authorities (Filippova and Noy 2020). Table 2 summarizes the evolution of seismic retrofit policy in New Zealand.

#### 2.2.2. Mechanisms for Financing Seismic Risk Mitigation

New Zealand established the Heritage Earthquake Upgrade Incentive Programme (EQUIP) in 2016 to support earthquake strengthening work for heritage buildings. Owners of heritage buildings can receive a grant from EQUIP that covers up to 50% of retrofit cost. Some local authorities, such as the Cities of Dunedin, Auckland, and Wellington, offer additional incentive funds to non-heritage buildings, but such assistance programs only exist in large cities where building owners are relatively wealthy and able to pay retrofit costs. This leads to a high retrofit rate in cities, but a low rate in small towns as they cannot afford strengthening costs (Filippova and Noy 2020). Thus, national incentives are crucial to enhancing the seismic resilience of small towns.

### 2.3. Japan

Many cities in Japan are located on coastal plains formed by sandy sediments and clay soil deposits at the mouths of rivers, which are weak and tend to liquefy during earthquakes. The Tokyo metropolitan area, the capital of Japan, is located on three layers of plates that rub or collide frequently, causing massive earthquakes. The probability of a magnitude 6 or stronger earthquake occurring in the next 30 years is 45 to 70% for the Tokyo metropolitan area (Headquarters for Earthquake Research Promotion 2018).

Japan has uniform national building standards implemented by both the national and local governments. Building standards are defined under the Building Standard Law, which facilitates compliance and control and ensures minimum specifications for safety (Tomohiro 2013). This is different from the United States, where building standards are developed by non-governmental technical associations, to be modified and enforced by states and jurisdictions (World Bank 2018).

In 1971, Japan amended the Building Standard Law and improved seismic design standards for reinforced concrete structures in response to the 1968 Tokachi Earthquake (M 8.3). Shortly thereafter, the 1978 Miyagi Earthquake (M 7.7) hit the northern region of Japan and caused the collapse of many buildings constructed under the 1971 Building Standard Law. In response, Japan modified design standards to not only prevent the collapse of buildings but also to secure the safety of people inside buildings during earthquakes, which enabled buildings constructed after 1981 to resist earthquakes that are one level higher (M 6) compared to previous requirements (M 5).

#### 2.3.1. Seismic Risk Mitigation Policies

Retrofit policy in Japan targets buildings by occupancy. The catalyst for modern retrofit policy was the 1995 Great Hanshin-Awaji Earthquake (M 6.9), which was centered in the southern region of Japan and resulted in 6,437 deaths and the collapse of 100,000 houses. A post-earthquake study reported that 76% of the collapsed buildings were constructed before 1971 and 21% between 1971 and 1981. This demonstrated that updating design standards alone is not enough to secure the safety of people and property. As a result, Japan adopted a set of provisions, including “The 1995 Act for Promotion of the Earthquake Proof Retrofit of Buildings” and “The 1998 Act for Support for Reconstructing Livelihoods of Disaster Victims,” to promote and support seismic retrofit for buildings constructed before 1981 (Ando 2012).

The 1995 Seismic Retrofitting Promotion Law provided measures and guidelines for identifying and strengthening seismically deficient existing buildings. The Law also contained retrofitting targets devised by the national government, such as raising the proportion of seismic-resistant houses and designated buildings (e.g., schools, hospitals) to 75% and 80% by 2003 and 2008, respectively. The Law was amended in 2005 to include the retrofitting promotion plans of local governments. Local governments tailored retrofitting targets to their conditions and needs, planned actions to be taken to achieve those targets, and designated emergency routes and evacuation facilities for their territories. In addition, the Ministry of Education, Culture, Sports, Science & Technology (MEXT) established policies for the structural and nonstructural retrofit of schools in Japan (Ando 2012). The Law was further amended in 2013, mandating seismic diagnosis for large-scale buildings such as hospitals, hotels, schools, and commercial facilities, and requiring that the diagnosis results be made publicly available. Table 3 summarizes the evolution of seismic retrofit policy in Japan.

The procedure for determining whether to retrofit or demolish a building in the diagnosis stage is as follows. A building that has an earthquake-resistant capacity equivalent to or above 60% of the requirement for new construction does not require strengthening. The decision to retrofit is affected by preservation needs, the target level of retrofitting, the availability of retrofitting methods, the costs of retrofit and demolition, the level of satisfaction with the building’s current functions and facilities, and the plan for future use of the building.

#### 2.3.2. Mechanisms for Financing Seismic Risk Mitigation

**Figure 1.**
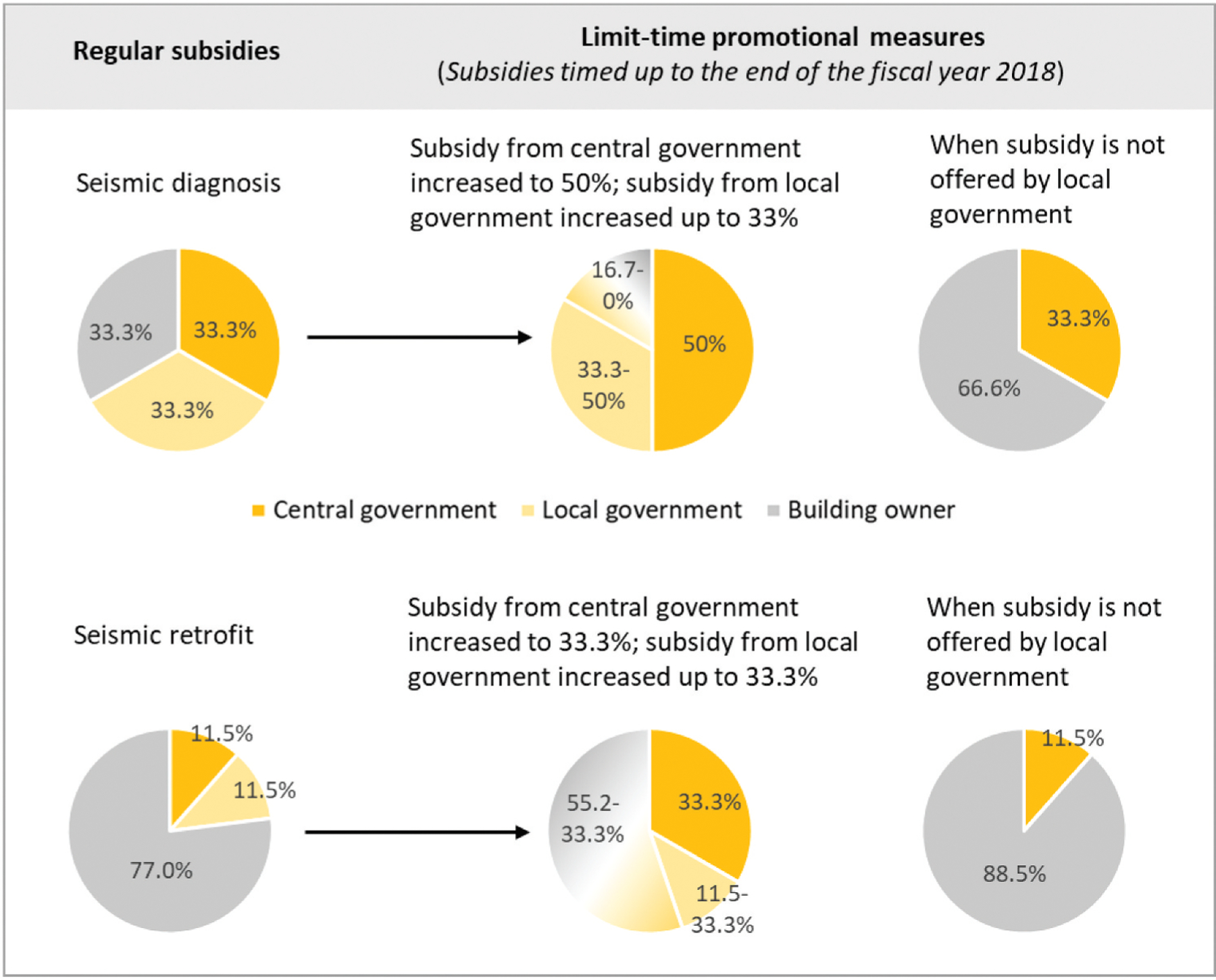
Subsidy coverage for seismic diagnosis and retrofit as a share of the required cost in Japan. Adapted from World Bank (2018).

Japan has implemented a system of financial incentives to encourage buildings owners to carry out needed retrofit measures (see Fig. 1). For detached houses, the subsidy of the central government and local governments covers 66.6% of seismic diagnosis cost and 23% of the seismic retrofitting cost, and homeowners pay the rest of the expenses. In addition, limited-time promotions were offered to houses retrofitted before the year 2019: the central and local governments offered an 83.4 to 100% subsidy for seismic diagnosis and a 45.8 to 66.7% subsidy for seismic retrofit. In the case that local governments did not offer any funding, the central government was responsible for 33.3% of diagnosis cost and 11.5% of retrofitting cost (World Bank 2018). Finally, local governments had access to subsidies and additional funding for the seismic assessment and retrofit of schools (Ando 2012).

Japan conducts various surveys to acquire data for formulating housing-related policy measures, including the Housing and Land Survey, Building Dynamic Statistics Survey, Comprehensive Survey of Living Conditions, and Housing Market Trend Survey (World Bank 2018). For instance, the 1995 and 2005 Seismic Retrofitting Promotion Laws mandated the government to raise the proportion of earthquake-resistant houses to 75% by 2003, 80% by 2008, and 90% by 2015. The 2008 Housing and Land Survey identified a 2% gap between the planned target rate and the actual rate, which prompted the government to devise policies to close the gap.

In addition to promoting building owners to comply with current building standards, Japan provides financial incentives for meeting higher than the mandatory standard. The Government Housing Loan Corporation, which provides long-term, low-interest loans for new construction, renovation, and retrofit, has established technical criteria beyond the mandatory minimum standard of the Building Standard Law. This enables buildings to achieve higher structural performance in a sustainable way, because enhanced construction quality triggers additional financing from private banks, and additional financing can fill funding gaps to ensure the completion of construction.

### 2.4. Italy

Italy faces substantial seismic risk due to its position in the Mediterranean where the African and Eurasian tectonic plates converge (Protezione Civile 2021). From 1905 to 2016, Italy experienced fifteen major earthquakes. The 2009 L’Aquila Earthquake (M 6.3) claimed more than 300 lives, and 10,000 buildings were damaged in the L’Aquila and Abruzzo regions (Georgescu et al. 2018). The 2016 Central Italy Earthquake Sequence (between August and Octorber 2016) resulted in 299 fatalities, 386 injuries, and nearly 5000 people were left without homes in the regions of Abruzzo, Lazio, Marche, and Umbria (Fiorentino et al. 2018).

The first seismic provision in Italy was issued in 1909 after the great Messina Straits Earthquake. The provision was revised several times, and the 1974 version can be considered the first modern building code. After the 1980 Irpinia Earthquake (M 6.9), a new seismic classification method was introduced into the seismic provision. Based on this method, about 45% of the Italian territory was classified in Seismic Zones 1, 2, and 3.

#### 2.4.1. Seismic Risk Mitigation Policies

Italy has one of the world’s oldest building stocks, with a large number of unreinforced masonry (URM) and reinforced concrete (RC) buildings. In the northern cities of Concordia della Secchia and Mirandola, for example, URM buildings account for about 87% and 70% of the building stock, respectively (Basaglia et al. 2020). Such buildings were not designed according to modern seismic codes and are subject to substantial earthquake risk. For instance, since compliance with the seismic provisions was not mandatory until 2009, older RC buildings have important structural deficiencies and need to be strengthened (Georgescu et al. 2018).

The first seismic code addressing existing buildings was published in 1986, and the code did not change substantially until 2003, when a new code based on Eurocode 8 was drafted and enacted. Eurocode 8 (EN 1998) is a harmonized technical rule that applies to the design and construction of buildings and civil engineering works in seismic regions. The code consists of six parts dealing with different types of construction, with EN 1998–3 providing technical standards for the seismic assessment and retrofit of existing buildings. The code defines basic performance requirements for structures under three limit states (near collapse, significant damage, and damage limitation), and provides compliance criteria including ensuring that the demands do not exceed corresponding capacities at each limit state. The code also depicts the information needed and the methods used for structural analysis, as well as safety verification criteria for reinforced concrete structures (Pinto and Franchin 2011).

The 2008 update introduced the “local strengthening” method for both public construction and private buildings to enable strengthening single structural elements or portions of a structure, without varying the global structural behavior. Moreover, a new seismic hazard map created using the micro-zonation method was adopted for a better identification of seismic hazards (Dolce 2012; Georgescu et al. 2018). Initially, only public strategic buildings (e.g., hospitals, schools) and infrastructure (e.g., highways, railways) were required to comply with the seismic code at the time. After the 2009 Abruzzo Earthquake (M 6.3), the seismic code was enforced for all types of construction.

Shortly after the enactment of the 2003 seismic code, Ordinance No. 3274 required local governments to complete seismic safety verification for all public strategic buildings and infrastructure in medium and high hazard regions within five years. The Law 326, enacted the same year, allocated a fund of €200 million to support the verification and seismic retrofit for buildings found deficient. By 2012, the number of verified buildings was far short of the requested 35,000, and thus the government decided to use the list of buildings to be verified to support the ongoing National Seismic Prevention Program (Dolce 2012). The major reason for the relatively slow verification process is that determining the seismic class of a building (see Table 4 below) requires field inspection, material testing, and structural simulation, which are time consuming (Cosenza et al. 2018).

#### 2.4.2. Mechanisms for Financing Seismic Risk Mitigation

After the 2009 Abruzzo Earthquake, the Law 77 granted €1 billion to the Department of Civil Protection for a seven-year National Seismic Prevention Program. The program funded seismic micro-zonation studies, retrofitted or reconstructed public strategic buildings and infrastructure, strengthened or reconstructed private buildings that are used by a large number of people, and developed emergency management tools and plans for urban communities (Dolce 2012). By 2018, a total of 4,521 interventions of public and private buildings had been funded and 1,249 had been completed, at a cost of €665 million (Protezione Civile 2021).

In 2017, Italy adopted the “Sismabonus” (or “quake bonus”), a tax incentive policy to motivate building owners to strengthen their buildings (Basaglia et al. 2020). The policy allows a tax reimbursement up to 85% of the retrofit cost for structural and nonstructural components, depending on the degree of improvement in the seismic class of the building, with deduction equally distributed over five to ten years (Cosenza et al. 2018). The seismic class is determined using either the expected annualized loss (EAL) approach or the safety index (IS-V) approach, as shown in Table 4. The EAL method calculates the ratio of direct economic loss, due to earthquake-related damages and repair costs for structural and nonstructural elements, to reconstruction cost. The IS-V method computes the ratio between designed and demanded peak ground accelerations for the life safety limit state. The safety threshold commonly used in reconstruction processes is IS-V at 60% (Caterino and Cosenza 2018; Cosenza et al. 2018).

The Italian government grants tax reimbursements to building owners as follows:

- 70% for independent/detached houses or manufacturing buildings that improve one class of risk;
- 80% for independent/detached houses or manufacturing buildings that improve two or more classes of risk;
- 75% for apartments that improve one class of risk;
- 85% for apartments that improve two or more classes of risk.

The amount of tax reimbursement cannot exceed 96,000 Euros for each real estate unit (Caterino and Cosenza 2018). While communities have demonstrated an interest in seismic risk assessment and mitigation, they often lack the necessary information and tools to understand the benefits of seismic strengthening and, in turn, of the “Sisma-bonus” (Basaglia et al. 2020).

Finally, Italy also encourages building owners to improve energy efficiency along with the seismic upgrade of existing buildings (Georgescu et al. 2018). Similar to the “Sisma-bonus,” the “Ecobonus” is a tax reimbursement for energy efficiency upgrades of up to 65% over ten years (Formisano, Vaiano, and Fabbrocino 2019). While the policies are not integrated, there is potential benefit to society from a combined policy. For instance, Formisano, Vaiano, and Fabbrocino (2019) present a global performance index that accounts for seismic, energy, and economic benefits from a combined policy. Table 5 summarizes the evolution of seismic retrofit policy in Italy.

### 2.5. China

Like Japan, China defines building standards under the Law to enforce compliance. The first seismic design standard (GBJ 11–89) came into effect in 1989. The standard was updated every ten years with minor amendments in between. Between the 1950s and 1980s, a large number of masonry and reinforced concrete buildings were constructed, designed to resist gravity loads without considering the effect of seismic loads. Today, those older buildings not only fail to meet seismic requirements, but are also subject to degradation due to aging and are unfavorable for energy conservation (Pan and Shan 2019).

China is one of the most seismically active countries in the world, as it lies on multiple active tectonic plates. The most devastating earthquake in recent decades, the 1976 Tangshan Earthquake (M 7.6), hit the most densely populated region of the country. More than 242,000 people died, 85% of buildings collapsed or were unusable in the City of Tangshan, and 10% of buildings collapsed in the capital, Beijing. In response, Beijing established an Earthquake Risk Reduction Council to repair damaged buildings and strengthen vulnerable structures in its district (Pan and Shan 2019). More recently, China amended the seismic design standard to ensure the sufficient capacity of schools and other critical buildings that can resist earthquakes of a higher magnitude, in response to the 2008 Wenchuan Earthquake (M 7.9).

#### 2.5.1. Seismic Risk Mitigation Policies

Similar to Japan, retrofit policy in China targets buildings by occupancy and potential earthquake risk. In China, the Department of Building and Development is responsible for building regulation and code enforcement. The Department issues the National Plan for Earthquake Hazards Reduction every five years. The 2016 Plan included the objectives of advancing the regulatory system, identifying high-risk buildings, retrofitting or reconstructing hazardous public buildings and residential buildings, strengthening heritage buildings, using informative tools to support seismic risk management, and promoting the development and deployment of earthquake-resistant techniques (Pan and Shan 2019).

#### 2.5.2. Mechanisms for Financing Seismic Risk Mitigation

In China, the cost for seismic retrofit, as well as the cost for improving building energy efficiency and upgrading non-structural components, is fully funded by local governments. Buildings are retrofitted based on government plans and budgets, which saves building owners time and money in submitting retrofit plans and applying for building permits. However, this policy greatly increases the financial burden of governments and hence has been implemented in a limited number of communities. By the end of 2017, Beijing had retrofitted more than two million square meters (21 million square feet) of housing (Pan and Shan 2019).

In 2016, the Chinese central government initiated a five-year plan to improve the seismic safety of high-risk buildings across the country. Since the plan was drafted at the national level, it allowed for allocation of funding and resources to urgent projects, such as buildings that were most vulnerable to earthquakes, or under-developed areas where local governments could not afford large-scale retrofitting (Pan and Shan 2019). Moreover, the plan allowed for coordination across different departments, construction sectors, and occupants, which greatly improved the efficiency of the retrofit work. However, it also increased the burden on local governments and discouraged building owners from playing an active role in enhancing seismic safety.

### 2.6. Iran

Iran is located on the active Alpine Belt. From 1990 to 2005, Iran experienced 14 earthquakes of magnitude 7.0 or above and 51 earthquakes of magnitude 6.0 to 6.9, which claimed more than 180,000 lives in total. Adobe and masonry buildings are the major type of structure in Iran and suffered the highest level of damage in historical earthquakes (Mostafaei and Kabeyasawa 2004). After the 1991 Manjil Earthquake (M 7.7), Iran established the Earthquake Hazard Mitigation Program, intended to advance scientific knowledge about earthquake hazard reduction, reduce the risk of structural failure and build safe structures, increase public awareness of seismic hazards, and develop plans for post-earthquake actions (Ghafory-Ashtiany, Jafari, and Tehranizadeh 2000).

The first Iranian Code of Practice for Seismic Resistant Design of Buildings was published in 1988, also known as Iran national standard No. 2800. The mandatory code has been updated three times, and the current version was released in 2015. Post-earthquake studies indicated that the damage rate of buildings constructed after 1990 is significantly lower than that of older buildings (Mostafaei and Kabeyasawa 2004). In 1997, the Earthquake Committee of the Iran Research Institute initiated a three-year earthquake research program to develop a comprehensive disaster management plan and earthquake resistant techniques for critical buildings and lifelines, involving more than 20 research institutes and universities across 78 projects (Ghafory-Ashtiany, Jafari, and Tehranizadeh 2000). The government’s collaboration with research institutes and universities to develop hazard mitigation plans, seismic design standards, and seismic retrofit guidelines, has enabled standards and policies to reflect state-of-the-art technology. This in turn prompted the development of seismic assessment methods and earthquake-resistant technologies to support building standards.

#### 2.6.1. Seismic Risk Mitigation Policies

Iran established a national earthquake hazard mitigation program in 2000, aiming to improve the resilience of critical buildings and lifelines across the country. The main objective of the “Doable Initiative and Momentum for Earthquake Hazard Reduction” was achieving seismic safety in Iran by 2020 (Ghafory-Ashtiany, Jafari, and Tehranizadeh 2000). In 2003, the Iranian government published a report entitled “Studying and Performing Retrofit for Critical Buildings and Lifelines,” which assessed the seismic vulnerability of seven structural groups. In addition, the government collaborated with the International Institute of Earthquake Engineering and Seismology to publish the “Instruction for Seismic Rehabilitation of Existing Buildings,” which provided guidelines and technical standards for seismic retrofit (Mahdi and Mahdi 2013).

In Iran, most retrofit activities occur for critical structures (e.g., nuclear facilities and school buildings), whereas older residential buildings are typically demolished and reconstructed because retrofitting is time-consuming and costly (Mahdi and Mahdi 2013; Raissi et al. 2014). In 2004, Iran assessed the vulnerability of 380,000 classrooms in 100,000 schools, estimating that 135,000 classrooms need to be reconstructed, and 126,000 classrooms require seismic upgrade.

#### 2.6.2. Mechanisms for Financing Seismic Risk Mitigation

In 2007, the Iranian Parliament granted 4 billion U.S. dollars to reconstruct or retrofit those hazardous schools (Raissi et al. 2014). The Housing Foundation, a semi-governmental organization, is responsible for the rehabilitation and construction of houses in rural areas as well as reconstruction of rural areas after a natural disaster. The organization helps building owners with financing, architecture and engineering services, construction materials and contractors.

### 2.7. Turkey

Turkey is located in a seismically active area where the Eurasian Plate collides frequently with the African and Arabian Plates. Two major fault lines, the North Anatolian Fault and East Anatolian Fault, pass through the country. In the last century, Turkey endured 111 earthquakes with a magnitude of 5 or more, and 55 earthquakes of a magnitude greater than 6.8 occurred between 1932 and 1999 (Gurenko et al. 2006). The 1999 Kocaeli Earthquake (M 7.4) led to 17,000 fatalities and 44,000 injuries and caused the collapse of approximately 20,000 buildings (Gurenko et al. 2006). The 2011 Van Earthquake (M 7.2) resulted in 600 deaths and an economic loss of almost $2 billion (Gunes 2015).

The first national seismic design code was published in 1944 after the 1939 Erzincan Earthquake (M 7.9), and the 1998 version can be considered the first modern seismic code. The Construction Inspection Law passed in 2001 led to better quality control of construction. Therefore, buildings constructed before 1980 are most vulnerable and need to be seismically upgraded, buildings constructed between 1980 and 2000 have a low risk of collapse during earthquakes, and buildings constructed after 2000 are considered to have sufficient earthquake-resistant capacities (Gunes 2015). It was estimated that about 23.4% of housing units were constructed in or before 1980, 43.5% in 1981–2000, and 21.8% in or after 2001 (TUIK 2011). The proportion of reinforced concrete buildings increased from one third in 1984 to nearly half in 2010, and more than 90% of buildings constructed in 2000–2010 were made of reinforced concrete (TUIK 2011).

#### 2.7.1. Seismic Risk Mitigation Policies

Until the 2011 Van Earthquake, Turkey’s seismic policy for existing buildings relied on post-earthquake recovery insurance and thus was largely reactive. The National Earthquake Insurance Program, established after the 1999 Kocaeli Earthquake, built a two-level earthquake insurance system to transfer risks via financial methods. Level one is the national compulsory earthquake insurance, called Turkish Catastrophe Insurance Pool (TCIP) and developed under the guidance of the World Bank to addresses structural damages. Level two is a private homeowner’s earthquake insurance, which covers structural, non-structural, and business interruption losses that exceed the TCIP’s limit. The premiums of the two levels of insurance are fixed by the government, and all companies in the market with a valid license are allowed to sell TCIP and private homeowners’ insurances (Durukal, Erdik, and Sesetyan 2006). The coverage of TCIP is limited to residential buildings and commercial units located in residential buildings, excluding post-1999 buildings without a legal construction permit. Losses caused directly or indirectly (e.g., fire, landslide) by earthquakes are counted with a coverage rate of 30% (Gurenko et al. 2006). Previous earthquake experience has demonstrated the remarkable performance of TCIP in helping victims quickly get back to normal life, yet there is a big concern about the capacity of TCIP in addressing a large number of claims after a major earthquake (Durukal, Erdik, and Sesetyan 2006).

After the 2011 Van Earthquake, the Turkish Parliament adopted the Urban Transformation Law, which required the Ministry of Environment and Urbanization (MEU) to identify and retrofit hazardous buildings in high-seismicity regions. The law established technical standards and procedures for seismic assessment, retrofit, and reconstruction of existing buildings, based on EN1998–3. Local technical commissions were formed, comprising ministry employees and professors in colleges, to evaluate and determine whether a building is hazardous.

#### 2.7.2. Mechanisms for Financing Seismic Risk Mitigation

In 2005, Turkey initiated the Istanbul Seismic Risk Mitigation and Emergency Preparedness Project (ISMEP), intended to enhance emergency preparedness, enforce building codes, and to mitigate seismic risks for critical public facilities. This project was funded by loans provided by several international funding institutions (Gunes 2015). About 283 school buildings and 3 health buildings have been reconstructed, and 796 school buildings and 48 health buildings have been retrofitted under ISMEP so far (IPKB 2020).

Turkey’s Urban Transformation initiative is partially funded by the European Investment Bank. According to official estimates, about 9 million buildings have insufficient seismic resistance capacities, which constitutes one third of the entire building block in Turkey. Retrofitting or reconstructing those buildings would cost $500 billion and need at least 20 years (Gunes 2015). For buildings at high risk, the MEU has the authority to evacuate and demolish the buildings without the consent of occupants. The MEU has also offered rent assistance to occupants during the reconstruction or retrofit process. The 2010 Turkish Seismic Code provided guidelines for rapid screening, structural evaluation, and strengthening of existing buildings, which helps speed up seismic upgrade for older buildings (Gunes 2015). Ultimately, retrofit cost is primarily the responsibility of the building owner.

### 2.8. Romania

Romania is periodically subject to strong earthquakes. The 1940 Vrancea Earthquake (M 7.7) caused considerable damage to masonry buildings in the southeast of the country. The tallest reinforced concrete building (a 12-story residential building) in the City of Bucharest collapsed entirely. After the earthquake, Romania adopted a tentative earthquake-resistant design code. The code was enacted in 1942, but the design code was not endorsed as a mandatory regulation until 1963 (Georgescu et al. 2018).

The 1977 Vrancea Earthquake (M 7.4) led to the collapse of 28 high-rise buildings that were slightly damaged in the 1940 earthquake. In addition, 156,000 apartments in urban areas and 21,500 buildings in rural areas were severely damaged or collapsed, and 366,000 urban apartments and 117,000 rural houses had to be repaired. About 1,578 people died, and more than 11,300 people were injured. After the earthquake, Romania updated the seismic zonation map to raise the seismic-resistant level of new construction (Georgescu et al. 2018) and introduced ductility rules for reinforced concrete structures, developed by the American Concrete Institute, into the Romanian Building Code (Vacareanu et al. 2004).

#### 2.8.1. Seismic Risk Mitigation Policies

Romania planned to strengthen 122 buildings designated as the most vulnerable structures in Bucharest (the capital and commercial center of Romania) following the 1977 earthquake. However, due to lack of experience in seismic retrofit, the Romanian government requested technical assistance from Japan in 1978, aiming to transfer Japanese seismic technology to its country (Vacareanu et al. 2004).

Following the 1986 and 1990 Vrancea Earthquakes (M 7.1 and M 6.9), the 1992 Romanian Building Code introduced a quantitative assessment approach, based on the seismic safety factor *R* (a parameter for a building’s ability to dissipate energy) to assist decision making for rehabilitation. In the mid-1990s, Romania started to incorporate European regulations into its building code. The 2008 Romanian Code for seismic assessment and retrofitting of existing buildings included the notions and concepts of European Standard EN 1998–3. The code also preserved the quantitative assessment approach based on the seismic safety factor and a three-tier approach, which is similar to that of the ASCE 31–02 standard, formerly FEMA-310.

#### 2.8.2. Mechanisms for Financing Seismic Risk Mitigation

In 1992, the Romanian government initiated a National Seismic Assessment and Rehabilitation Program for existing buildings. The government fully funded seismic assessment and regularly published the list of assessed buildings and their risk classes. For buildings identified to be at risk, owners were responsible for retrofit cost. The government offered a low-interest loan to motivate building owners to strengthen their buildings (Craifaleanu 2013; Georgescu et al. 2018). By 2013, most buildings were seismically assessed, but only a few conducted retrofit work. The major reasons are that apartment buildings (dominant building type in Romania) with many owners cannot reach an agreement for retrofit; most occupants are reluctant to move to temporary houses; and building owners are concerned about retrofit cost (Craifaleanu 2013).

### 2.9. India

India is prone to devastating earthquakes caused by the collision of the Indian Plate and the Eurasian Plate, which has formed the Himalaya Mountains. Seven earthquakes between 1988 and 2005 of M 6.0 or greater claimed more than 25,000 lives and rendered approximately a million houses uninhabitable in India. Over 95% of the fatalities were found in non-engineered houses, which were constructed in a traditional manner without the design of qualified architects and engineers (National Disaster Management Authority 2014).

India published the first seismic code (IS 1893) in 1962, which was not mandatory until 2001. In 1991, the code was split to address four structural groups (buildings, liquid retaining tanks, bridges and retaining walls, and industrial structures). Two new codes for non-engineered construction (IS 13827 and IS 13828) were published in 1993, providing seismic provisions for earthen buildings and low-strength masonry buildings, respectively. The two codes were intended to improve the seismic performance of non-engineered structures rather than to stop the construction of such buildings (Jain 2016).

About 95% of buildings in India are made of mud, stone, bricks, timber, and bamboo, while concrete or steel structures constitute 3.6% of entire building stock (National Disaster Management Authority 2014). Reinforced concrete is used in urban and suburban areas for multi-story buildings. In the 2001 Bhuj Earthquake (M 7.7), about 130 modern reinforced concrete buildings collapsed in the City of Ahmedabad, whereas government-owned buildings designed under the seismic code were not damaged. This is attributable to lack of systematic code enforcement in private buildings and the shortage of appropriately trained civil or structural engineers in the country (Jain 2016). After the 2001 earthquake, many cities and states began to require compliance with seismic codes; however, they did not take measures to enforce such compliance.

A national Earthquake Engineering Training program was implemented during 2003–2007 by the Indian Institutes of Technology and the Indian Institute of Science, with financial support from the government. The program offered training to more than a thousand teachers in Civil Engineering and Architecture colleges, which enabled the concepts and techniques of seismic engineering to be taught in the classroom (Jain 2016).

#### 2.9.1. Seismic Risk Mitigation Policies

The code for seismic evaluation and strengthening of existing buildings (IS 15988) was published in 2013. In 2014, the Indian government published the National Disaster Management Guidelines on Seismic Retrofitting of Deficient Buildings and Structures, which recommended making seismic retrofit mandatory for existing government-owned buildings and privately-owned buildings that are critical to industrial and commercial activities, safety and security, health care, and education. The guidelines also encouraged the retrofit of (1) buildings located in high-risk seismic zones, (2) unreinforced masonry buildings and reinforced concrete buildings with open ground floors or soft stories, and (3) buildings not designed by appropriately credentialed engineers or constructed based on improper designs. Moreover, a cost-based decision-making method was provided for building owners to determine whether to retrofit or reconstruct their buildings, as described in Table 6 (National Disaster Management Authority 2014).

#### 2.9.2. Mechanisms for Financing Seismic Risk Mitigation

The National Disaster Management Authority (2014) calls for further research into the feasibility of offering long-term loans with low interest rates to support seismic retrofitting. Moreover, funds are available through various programs for government-owned buildings, which have been prioritized for retrofit. However, the priority in India is ensuring that new constructions comply with seismic codes, which is seen as cheaper and more effective than retrofit (Jain 2016). Thus, retrofit cost is primarily the responsibility of the building owner.

### 2.10. Nepal

Nepal lies on two active tectonic plates, the Indian Plate and the Tibetan Plate, which have produced frequent, devastating earthquakes. The 1988 Nepal Earthquake (M 6.9) killed 721 people and destroyed more than 7,000 buildings. The 2015 Nepal Earthquake (M 7.8) caused the collapse of 500,000 residential buildings and 2,656 office buildings, with an additional 200,000 residential buildings and 3,622 office buildings damaged. An estimated 8,790 people died and 22,300 people were injured (Shrestha, Subedi, and Rajbhandari 2016).

The National Building Code was adopted in 2003, providing the first seismic design standard for new buildings in Nepal. However, the code was not enforced effectively because monitoring the implementation of the regulation proved challenging. Some municipalities around Kathmandu Valley, the most developed and populated region in Nepal, allowed voluntary compliance with the code (Nepal Earthquake Clearinghouse 2015). In general, buildings constructed after 2004 are considered to be “seismically safe.” In Nepal, early structures were mostly built of adobe and low strength masonry, which are susceptible to earthquakes and many other natural factors (e.g., drying shrinkage, thermal movements, foundation settlements, plant growth). However, some of those structures are historically significant and culturally important to preserve (Shrestha, Subedi, and Rajbhandari 2016).

Over the last two decades, the United Nations Development Programme (UNDP) assisted the Government of Nepal in preparing the National Building Code, developing curricula and manuals for engineers and masons to properly implement the Building Code, and establishing code-compliant building permit systems in several municipalities. In 2011, the UNDP launched the Comprehensive Disaster Risk Management Programme (CDRMP) to improve resilience and strengthen the institutional and legislative sectors of Nepal for disaster risk management. This involves building the capacity of government ministers, departments and agencies, and local governments, and strengthening the partnership between governments and private sectors (Shrestha, Subedi, and Rajbhandari 2016).

#### 2.10.1. Seismic Risk Mitigation Policies

After the 2011 Sikkim Earthquake (M 6.9), the UNDP, in collaboration with Nepal’s Department of Urban Development and Building Construction and Center of Resilient Development, developed the first seismic retrofit guidelines for three dominant building categories: adobe, masonry, and reinforced concrete structures. After the 2015 Nepal Earthquake (M 7.8), the Government of Nepal realized the importance and urgency of strengthening existing buildings and adopted the seismic retrofit guidelines (Shrestha, Subedi, and Rajbhandari 2016). The guidelines provide the methods for vulnerability analysis, multiple options for structural strengthening, and the minimum requirements for seismic performances of retrofitted buildings.

#### 2.10.2. Mechanisms for Financing Seismic Risk Mitigation

Nepal’s National Society for Earthquake Technology (NSET), with the support of the Asian Development Bank (ADB), developed a five-year plan to strengthen 900 schools in the Kathmandu Valley at an estimated cost of $30 million. Funding was provided by the national and local governments, the ADB, Aus Aid, the government of Japan, and the World Bank (Vishokarma et al. 2012). In addition, NSET and the United Nations Centre for Regional Development (UNCRD) have conducted training programs to educate female heads of household about residential non-structural mitigation, such as securing refrigerators and shelves (Ando 2012). Financial support for the seismic retrofit of privately owned buildings is not available, but the Government of Nepal recommends retrofitting if the cost is below 25% of the replacement cost (Shrestha, Subedi, and Rajbhandari 2016).

## 3. Recommendations

The key takeaway that emerges is that adoption of retrofit policies has historically been driven by earthquake impacts. Major earthquakes have been followed by significant policy changes that seek to protect life and property. Countries in this study have recently experienced, or continue to experience, destructive earthquake events while the United States has not endured one since Northridge in 1994.

National, state, and local governments need not to wait for another catastrophe to mitigate risk from seismic impacts. Engineering and implementation knowledge necessary for effective retrofit interventions is available and can prevent many deaths and injuries in inevitable future earthquake events. For example, from Japan comes the knowledge that rigorous monitoring and regular policy updates are essential. Japan not only amends building regulations after an earthquake to prevent structural defects in new construction, but also conducts periodic surveys such as the Housing and Land Survey to formulate housing-related policies to enhance seismic resilience. The government also adjusts incentive policies to close a critical gap between planned and actual rates of retrofit (World Bank 2018).

Design standards and retrofit policies are adopted at the local level in the United States, which is very different from procedures in other countries. Nevertheless, lessons from other countries may prove useful to state and local governments attempting to enhance seismic resilience in the United States, as well as governments in any earthquake-prone country. Based on the lessons learned from the preceding comparative study of seismic retrofit policies, recommendations are organized around key stages of the seismic retrofit process. It is important to note that this process should not be seen as one-time or linear, but instead should be part of a recurring cycle, from risk assessment through monitoring, to continue improving the seismic resilience of the building stock. In this way, governments can continue to find appropriate solutions that will help prevent deaths, injuries, destruction, and downtime.

### 3.1. Recommendation 1: Risk Assessment

The first step in designing a retrofit policy is to identify the problem. Thus, seismic evaluation of buildings is seen to be an integral component of retrofit policies around the world. The lessons learned suggest the following best practices for assessing risk.

- Support for seismic evaluation: Japan’s Seismic Retrofitting Promotion Laws and Romania’s National Seismic Assessment and Rehabilitation Program provide generous subsidies to building owners for seismic evaluation, which is less expensive and thus easier for local governments to partially or fully fund than the retrofit itself (Georgescu et al. 2018; World Bank 2018). In addition, Romania and Turkey introduced new evaluation methods to their codes, enabling rapid screening and assessment for existing buildings (Georgescu et al. 2018; Gunes 2015). This suggests the need for constant improvement of evaluation guidelines and standards to provide more accurate and rapid and less expensive evaluation methods.
- Public results: Information about the potential risk posed by buildings should be available and accessible to the community, as in Japan and Romania. In the U.S., cities and counties in California with retrofit mandates identified properties that must be retrofitted and made lists publicly available so that building users and community members were informed about high-risk buildings in their community. In addition, data repositories should have procedures to record updated information regarding retrofit or reconstruction.
- Communicate results: It is imperative to explain risk from seismic events to non-technical audiences in a clear and simple way. Currently, the U.S. does not use a comprehensive mechanism to communicate to the public, even implicitly, a building’s anticipated performance or risk level in an earthquake event. Italy uses the seismic class to describe the risk level of a building (Cosenza et al. 2018). New Zealand’s Building Act employs the percent of new building standards (%NBS) to indicate seismic risk (Egbelakin 2013).
- Invisible risk: Buildings that have undergone some level of shaking should be evaluated even if no damage is apparent. A building that survives an earthquake could still result in substantial losses in a subsequent event. In Romania, buildings that survived an earthquake with only slight damage collapsed 37 years later in another earthquake (Georgescu et al. 2018).

### 3.2. Recommendation 2: Knowledge Transfer

Federal guidelines and provisions, together with the national model building code, often provide the foundation for local retrofit policy in the United States. Nevertheless, state and local governments may benefit from technical knowledge and support from experts in the field. In addition, information sharing regionally, nationally, and internationally is an important mechanism by which the field of earthquake risk mitigation evolves. The following best practices are identified:

- Decision-support tools: Communities and building owners can make informed decisions with the right tools. For example, India (National Disaster Management Authority 2014), Japan (World Bank 2018), and New Zealand (Egbelakin 2013) developed tools for making retrofit decisions that include cost evaluation. In the case of India, the cost is holistic and includes debris removal (see Table 6). While many such tools exist, a national standard for a cost-based retrofit decision tool would help communities develop, and building owners respond to, a retrofit policy.
- Public-private partnerships: Partnerships with the private sector and academia can be invaluable for expanding the local knowledge base, educating the public, and increasing technical training. In Turkey, seismic evaluation procedures under the Urban Transformation Law were developed and conducted collaboratively between ministry employees and scholars in the field (Gunes 2015). Such partnerships have also been an important part of retrofit policy in Iran (Ghafory-Ashtiany, Jafari, and Tehranizadeh 2000) and Nepal (Shrestha, Subedi, and Rajbhandari 2016. In the U.S., the city of Seattle partnered with a nonprofit to develop financing strategies for its retrofit program (NDC 2019). In addition, most code committees in the U.S. include experts from both academia and industry.
- Local knowledge: No one understands the risk to a community better than the community itself. In Italy, the Civil Protection Department educates the public on seismic, flood, and landslide risk during public awareness days called “I do not risk” (Protezione Civile 2021). Moreover, local knowledge may be useful to other governments facing similar risks, especially those without the resources to develop mitigation policy on their own. In the U.S., San Francisco’s soft-story retrofit mandate influenced the adoption of a similar mandate in other cities across California (City of San Francisco 013). Moreover, local knowledge can become global. For instance, Fremont’s soft-story retrofit standards formed the basis for the 2003 IEBC (City of Fremont 2007), while Romania turned to Japan for technical assistance for its retrofit program (Vacareanu et al. 2004).

### 3.3. Recommendation 3: Setting Priorities

Traditionally, the most vulnerable buildings have been prioritized and selected based on construction type; for example, unreinforced masonry buildings are well documented as some of the most vulnerable (Turner 2020). Beyond addressing the most vulnerable buildings, retrofit policies can focus on “critical” buildings for which failure would pose significant risk to public safety, based on their occupancy class and role in the community.

- Prioritize building use: In addition to targeting vulnerable buildings by construction type, policies can target building use. For instance, China’s National Plan for Earthquake Hazards Reduction, Iran’s Doable Initiative, and Japan’s Seismic Retrofitting Promotion Law target “critical” buildings such as hospitals and schools through either mandatory retrofits or incentives. It is also important for recovery, a primary motivation for the mandated retrofit of hospitals in California (Preston et al. 2019).
- Prioritize public buildings: Another important category that is often targeted by retrofit policies is that of government buildings (e.g., India’s National Disaster Management Guidelines on Seismic Retrofitting of Deficient Buildings and Structures and the Istanbul Seismic Risk Mitigation and Emergency Preparedness Project in Turkey). In the U.S., Presidential Executive Order (EO 13717) recommends that federal buildings be retrofitted to improve the seismic resilience of the nation, while San Francisco retrofitted over 100 of its public buildings.

### 3.4. Recommendation 4: Implementation

Perhaps the most challenging stage of the retrofit process is making sure a retrofit happens. Even under retrofit mandates, compliance is not always ensured. However, financial support has been shown to increase compliance rates. Another route to compliance is to bundle seismic retrofit with other upgrades for which a building owner is willing to pay. In many countries, including the U.S., buildings that sustain damage in an earthquake, as well as buildings that are being renovated, can trigger a mandatory retrofit. An extension of this idea leverages interest in sustainable infrastructure by pairing energy-efficiency upgrades with seismic retrofit. The lessons suggest the best practices for implementation of retrofit policy:

- Financial support: It is difficult to monitor and enforce retrofit standards on residential properties. Several countries thus offer financial support for voluntary residential retrofits, such as Japan’s system of subsidies (World Bank 2018) and Italy’s “Sisma-bonus” tax reimbursement (Cosenza et al. 2018), or government funds for mandatory retrofit activities, as in China (Pan and Shan 2019). Financial support and incentives can help building owners implement the policy. For example, Japan provided subsidies to building owners for seismic diagnosis and retrofit, which enabled the compliance rate to meet planned targets at each stage (World Bank 2018). On the other hand, not all financial support induces compliance. Romania offered low-interest loans to building owners for voluntary retrofit, which did not lead to increased retrofit activities (Craifaleanu 2013).
- Normalize retrofit procedures: Part of the reason that retrofit is so costly is because it is unique to each building. In China, buildings are retrofitted based on government plans and budgets, which saves building owners time and money in submitting retrofit plans and applying for building permits (Pan and Shan 2019). Similar efforts to standardize and normalize retrofit within the U.S. could be further explored.
- Triggers: a seismic retrofit can be conducted as a standalone process. However, it may be more effective when coupled with another activity already taking place and can therefore be administratively folded into existing work. New Zealand’s Building Act 2014 stipulates that structural alteration to a building’s function, including expanding rooms or adding new rooms, triggers a seismic retrofit. Similarly, in the U.S., alterations, additions, change of occupancy, and change of use may trigger a retrofit (Searer and Rosenboom 2014). One way to streamline the process is to require seismic evaluation when applying for a renovation permit, with all of the monitoring and paperwork going through the existing administrative and oversight processes. New Zealand’s Building Act 2016 tasked territorial authorities with identifying procedures by which to apply standards and requirements. Linking to in-place procedures will help leverage existing effort and investment, rather than requiring separate retrofit-oriented efforts.
- Integration with other objectives: Countries such as China (Pan and Shan 2019) and Italy (Formisano, Vaiano, and Fabbrocino 2019) pair seismic retrofits with energy-efficiency upgrades. Property Assessed Clean Energy (PACE) financing in the U.S. has been extended to include seismic retrofit of commercial properties in California, Oregon, and Utah (McKernon 2018), in an effort to incentivize seismic resilience paired with sustainability. In addition to sustainability, communities may be interested in climate-change adaptation. The European Union has developed policy and guidelines to support actions that reduce a building’s vulnerability to increased storms, snow, extreme temperatures, and other impacts associated with climate change (European Commission 2013). One can imagine a strategy that pairs seismic resilience with climate-change adaptation analogously to existing strategies that pair seismic resilience with sustainability.

### 3.5. Recommendation 5: Monitoring

Once a retrofit policy is in place, it is important to ensure that policies and activities are having their intended effect. Is seismic risk reduced? Can we reasonably assume that deaths, injuries, or downtime have been reduced? Policy development alone is insufficient to ensure impact. The lessons learned from other countries suggest that continuous monitoring and updating is a best practice:

- Monitoring and evaluation: Risk mitigation is not a one-time process. Regular monitoring and evaluation are necessary to ground-truth retrofit efforts. In addition to the collection of information at the time of retrofit construction application or completion, there are other methods by which to gather relevant information. For example, Japan conducts regular surveys to collect information on structures, assess impacts and deficiencies, and help to inform policy updates (World Bank 2018). Ensuring the adequate performance of the building stock is invaluable. Turkey has instituted measures for quality control on construction, with assessments that answer the question: “Do the buildings actually provide the level of performance that their designs dictate that they provide?” (Gunes 2015). The ability for a jurisdiction to reliably count on a building to protect lives after an earthquake event is crucial to policy success.
- Holistic perspective: Retrofit of individual building assets is incredibly important, particularly for building occupants, users, residents, or owners. However, building failure and collapse can have significant consequences not only for individuals associated with that building, but for those nearby, in addition to other cumulative consequences of building failure across the area, city, or region. Any jurisdiction looking to mitigate risk may want to consider what geographic or demographic areas of the city or region should be targeted for retrofit activities in order to ensure the best post-earthquake outcomes. Japan’s 2005 Seismic Retrofitting Promotion Law designated emergency routes and evacuation facilities specific to the earthquake hazard. These routes and infrastructure would have retrofit actions considered to ensure their usability. In Turkey, some buildings are considered to be so risky to the population that they are mandated for demolition, with the government providing rent assistance to displaced residents (Gunes 2015). The U.S. has had great success in utilizing scenarios to help visualize impacts to the built environment across regions and set the stage for risk mitigation planning (Hudnut et al. 2018; PNSN 2020).
- Assuring recovery: Knowing that retrofit policies take time to yield results, and that some kinds of buildings may not be suitable for retrofit, policy makers may also consider the utility of earthquake insurance. In the event of an earthquake, insurance can enable repairs or reconstruction, and help ensure community continuity that could be in danger following a large-scale earthquake with regional impacts. Turkey offers compulsory earthquake insurance for residential buildings, the Turkish Catastrophe Insurance Pool (TCIP). While this kind of requirement may not be appropriate in all areas of the U.S., for those places with significant risk, or with significant risk particularly from older buildings, mandatory earthquake insurance may provide some protection to communities and building owners in cases where retrofits cannot reasonably be applied, or older buildings are able to avoid retrofit triggers.

## 4. Conclusions

In countries that have proactive retrofit policies in place, including Japan, Italy, and New Zealand, the number of collapses and severely damaged buildings in recent earthquakes is lower than would have been expected. This suggests that improved seismic design, including retrofits, can have a meaningful impact on building performance during an earthquake event. The lessons learned suggest several recommendations for state and local governments designing seismic retrofit policy to improve their seismic resilience. The recommendations are organized around key stages of the retrofit process, with the understanding that risk mitigation planning is a nonlinear and recurring process. While the experiences of other countries with retrofit policy are instructive for the United States, several gaps remain.

Across the countries in the study, retrofit policies have largely targeted the most vulnerable buildings: masonry and adobe, older concrete, and wood-frame buildings, depending on their prevalence. Beyond addressing the most vulnerable buildings, retrofit policies tend to focus on “critical” buildings for which failure would pose significant risk to public safety. The question of how to prioritize buildings is highly context-specific to each country. There seems to be consensus around schools and hospitals, but some countries may consider buildings that host large numbers of people, such as high-rise office buildings, department stores, and hotels in Japan, while others may consider critical infrastructure, such as nuclear facilities in Iran. A potential direction for future research is to investigate and identify parameters for the spectrum of retrofit interventions, from minimum standards (focusing on those buildings posing the greatest risk) to the maximum application (going beyond which will provide little return or benefit).

Questions also remain about how to ensure compliance, especially under voluntary policies. Even under mandatory policies, compliance is not always 100%. Another obstacle is that not enough is known about retrofit cost, which tends to be variable across countries, and therefore, designing appropriate incentives is challenging. For the U.S., methods exist for estimating structural retrofit cost (e.g., Fung et al. 2020a), but there is comparably very little known about other costs, including non-structural retrofit cost (Fung et al. 2020b).

Finally, timing is an important concern. Several cities in the U.S. have set compliance timelines only to revise them due to low rates of compliance. Countries such as Japan continually update their targets by collecting data. The tradeoff between providing sufficient time for compliance and ensuring sufficient compliance rates has not been investigated in the literature.

## Figures and Tables

**Table 1.** Seismic retrofit policies of earthquake-prone countries.

Country	Typical vulnerable buildings	Retrofit policies	Mechanisms for financing retrofits
United States	Unreinforced masonry buildings, wood-frame soft-story buildings constructed before 1978, concrete buildings constructed before 1976, pre-Northridge steel moment frame buildings	**Policies vary at local government level** **California State**: mandatory retrofit for unreinforced masonry buildings in regions of the highest seismicity; mandatory retrofit for wood-frame soft-story buildings in San Francisco Bay area and Los Angeles metropolitan region; mandatory retrofit for concrete and steel moment frame buildings in Los Angeles metropolitan region. Some exceptions applied under each mandate **Oregon State**: mandatory retrofit for unreinforced masonry buildings in the Portland City with different requirements for Building Class I to V, voluntary retrofit for unreinforced masonry buildings in other regions **Washington State**: preparing a mandatory retrofit policy for unreinforced masonry buildings	Retrofit grants from the Federal Emergency Management Agency, California Earthquake Authority, or the City Council Fee waiver for plan review, building permits, or both Long-term loans with a low-interest rate, sponsored by the city government Pass retrofit cost to tenants
New Zealand	Unreinforced masonry buildings any buildings with three or more stories constructed before 1976, any buildings with one or two stories constructed before 1935	73% of territorial authorities adopting seismic retrofit levels between 33% and 67% of new building standards, and 27% of them approving 67%; 45% of local authorities choosing an active approach to implement retrofit policies, 32% of them using a passive approach, and 23% of them employing combined active-passive approach	Funded by the government if the building is recognized as a heritage
Japan	Masonry and concrete buildings constructed before 1981	Mandatory retrofit for large-scale buildings and public buildings, voluntary retrofit for small- and medium-size residential buildings	**Regular incentive**: governmental subsidy covering 66.6% of seismic diagnosis cost and 23% of the seismic retrofit cost **Limited-time promotion**: governmental subsidy covering 83.4–100% of diagnosing cost and 45.8–66.7% of retrofit cost
Italy	Unreinforced masonry buildings and reinforced concrete buildings constructed before 1980	Compulsory earthquake insurance for residential buildings	Sponsored or reinsured by the government
China	Masonry and concrete buildings constructed before 1989	Retrofit work planned and conducted by local governments	Fully funded by local governments
Iran	Adobe, masonry, and concrete buildings constructed before 1990	Voluntary retrofit for residential buildings	Supported by Iranian Housing Foundation
Turkey	Masonry and concrete buildings constructed before 1980 Masonry and concrete buildings constructed before 1980	Retrofit or reconstruction for schools, hospitals, and other priority public buildings Compulsory earthquake insurance for residential buildings	Financed by the World Bank, European Investment Bank, and Council of Europe Development Bank Sponsored or reinsured by the government
Romania	Buildings constructed before 1941, concrete buildings with three or more stories constructed before 1978	Voluntary retrofit for residential buildings	Supported by Romanian Ministry of Regional Development and Public Administration
India	Buildings constructed before 1962, concrete buildings with open ground floors or soft stories, non-engineered structures	Mandatory seismic retrofit for government-owned buildings and selected privately owned buildings (e.g., hospitals, schools), voluntary retrofit for other privately-owned buildings	Supported by Indian National Disaster Management Authority
Nepal	Adobe, masonry, and reinforced concrete buildings constructed before 2004	Retrofit or reconstruction for schools Voluntary retrofit for residential buildings	Financed by Asian Development Bank, World Bank, Australian aid Supported by Nepal Ministry of Urban Development

**Table 2.** The evolution of retrofit policy in New Zealand.

Year	Retrofit policy
2004	*Building Act 2004* Mandated territorial authorities to adopt a policy on earthquake-prone buildings within 18 months Required each territorial authority to provide a copy of the policy to the chief executive and adhere to the special consultative procedure when developing, amending, or replacing a policy Required territorial authorities to complete a review of a policy within 5 years after the policy is adopted and then at intervals of not more than 5 years Authorized territorial authorities to carry out seismic work (retrofit or demolition) if any work required under a notice given by the territorial authority is not completed, or not proceeding with reasonable speed
2016	*Building Act 2016* Mandated territorial authorities to identify potentially earthquake-prone buildings 15 years for an area of low seismic risk 5 years for priority building (e.g., hospitals, emergency centers, police offices, schools) and 10 years for other buildings for an area of medium seismic risk 2 years and 6 months for priority buildings and 5 years for other buildings for an area of high seismic risk Required building owners to complete engineering assessment for their potentially earthquake-prone buildings Required territorial authorities to determine whether a building is earthquake-prone and to issue a notice for earthquake-prone buildings Set deadlines for completing seismic work 35 years for any building in an area of low seismic risk 12 years and 6 months for a priority building and 25 years for any other building in an area of medium seismic risk 7 years and 6 months for a priority building and 15 years for any other building in an area of high seismic risk Granted exemption from compliance or an extension of time in some cases Authorized territorial authorities to assess information relating to earthquake-prone building status at any time, to impose safety requirements, and to carry out seismic work (retrofit or demolition)

Sources: The Parliament of New Zealand (2004) and The Parliament of New Zealand (2016).

**Table 3.** The evolution of seismic retrofit policy in Japan.

Year	Retrofit policy and targets (in bold)	Earthquake trigger
1995	*Seismic Retrofitting Promotion Law (Enforcement)* Provided measures and guidelines for diagnosing and retrofitting seismically deficient existing buildings Planned to raise the proportion of earthquake-resistant **houses** and **designated buildings** (e.g., schools, hospitals) to 75% by 2003 and 80% by 2008	The 1995 Hanshin-Awaji Earthquake (M 7.3)
2005	*Seismic Retrofitting Promotion Law (Amendment)* Included local government’s seismic retrofitting promotion plans: Measures for the efficient promotion of seismic-resistant buildings Designation of emergency routes and evacuation facilities Planned to increase the proportion of earthquake-resistant **houses** and **designated buildings** to 90% by 2015	The 2004 Nigata Chuetsu Earthquake (M 6.8)
2013	*Seismic Retrofitting Promotion Law (Amendment)* Mandated seismic diagnosis for **large-scale buildings** and **public buildings**: Large-scale buildings used by large numbers of people, such as hospitals, department stores, and hotels (within 2 years) Large-scale buildings used by people having difficulty in evacuating, such as younger school children and the elderly (within 2 years) Buildings along the emergency routes designated by prefectural or municipal governments (no time limit) Designated evacuation facilities and government buildings used as emergency operation hubs (no time limit) Required publishing or making publicly available the diagnosis results Planned to raise the proportion of earthquake-resistant **houses** to 95% by 2020	The 2011 Great East Japan Earthquake (M 9.0)

Buildings that meet the requirements of the 1981 Building Standard Law are deemed earthquake resistant. Sources: World Bank (2018), Hasegawa (2013), and Tomohiro (2013).

**Table 4.** Seismic classification based on the expected annual loss (EAL) and safety index (IS-V) approaches. Adapted from Caterino and Cosenza (2018).

EAL (%)	Seismic class by EAL	IS-V (%)	Seismic class by IS-V
Less than 0.5	$A_{EAL}^{+}$	Greater than 100	$A_{IS-V}^{+}$
0.5–1.0	*A*_*EAL*_	80–100	*A*_*IS*–*V*_
1.0–1.5	*B*_*EAL*_	60–80	*B*_*IS*–*V*_
1.5–2.5	*C*_*EAL*_	45–60	*C*_*IS*–*V*_
2.5–3.5	*D*_*EAL*_	30–45	*D*_*IS*–*V*_
3.5–4.5	*E*_*EAL*_	15–30	*E*_*IS*–*V*_
4.5–7.5	*F*_*EAL*_	Less than 15	*F*_*IS*–*V*_
Greater than 7.5	*G*_*EAL*_		

**Table 5.** The evolution of retrofit policy in Italy.

Year	Retrofit policy and targets (in bold)	Trigger
2003	*Ordinance No.3274 “Technical rules for the design, evaluation and seismic retrofitting of buildings”* Regulated the procedure for structural evaluation Defined the methods for seismic analysis| Provided instructions for building safety verifications Required local governments to complete seismic safety verification for all **public strategic buildings** (e.g., hospitals, schools) and **infrastructure** in medium and high hazard areas in five years *Law 326* Allocated a fund of €200 million to support building safety verification and seismic upgrade for **public strategic buildings** and **infrastructure**	The 2002 Molise Earthquake (M 5.9)
2008	*Italian Guidelines for Micro-zonation* Mandated the use of a new seismic hazard map (OPCM 3519/2006) for seismic upgrade design Introduced the “local strengthening” method for both **public constructions** and **private buildings** to enable strengthening single structural elements or portions of a structure, without varying the global structural behavior *Law 326* Allocated an annual fund of €200 million to support the seismic retrofit of **schools**	
2009	*Law 77* Mandated the use of 2008 standards for any kind of construction beginning on July 1, 2009 Allocated a fund of €1 billion in the next seven years to support the National Seismic Prevention Program	The 2009 Abruzzo Earthquake (M 6.3)
2010	*Ordinance 3843* Established a Commission of scientists and experts to define general objectives and criteria for seismic risk reduction Reducing the risk of life loss rather than economic loss Improving techniques and knowledge for seismic retrofit Requesting local government agencies and private sectors for co-funding	
2017	*Stability Law 2017* Provided tax reimbursement up to 85% of the retrofit cost, depending on the degree of improvement in the seismic class of the building, with deduction equally distributed over five to ten years	The 2016 Central Italy Earthquake (M 6.2)

Sources: Dolce (2012), Caterino and Cosenza (2018), and Georgescu et al. (2018).

**Table 6.** Cost-based decision making for seismic retrofit (Adapted from National Disaster Management Authority 2014).

Decision	Cost of seismic retrofit as a percentage of cost of reconstruction^1^	
	Critical and lifeline facilities	Office and residential facilities
Retrofit	If retrofit cost is less than 50% of reconstruction cost	If retrofit cost is less than 30% of reconstruction cost
Detailed technical assessment for retrofit^2^	If retrofit cost is between 50–70% of reconstruction cost	If retrofit cost is between 30–50% of reconstruction cost
Reconstruction	If retrofit cost is greater than 70% of reconstruction cost	If retrofit cost is greater than 50% of reconstruction cost

1 Reconstruction includes demolition, removal of debris, and construction.

2 The assessment determines vulnerability of the building or structure by analyzing the cost, age, heritage value, proximity to archaeological structures, criticality of the building, current and projected floor area ratios, residual life, potential disruption, expansion and upgradation of services, and potential for improved function.
